# Functional Impact of Pain and Its Association With Sarcopenia in the Elderly: The Birjand Longitudinal Aging Study (BLAS)

**DOI:** 10.1155/prm/7121728

**Published:** 2026-07-03

**Authors:** Shakiba Rahimi, Negar Zareshahi, Azin Soltani, Akam Ramezani, Hossein Fakhrzadeh, Masoumeh Khorashadizadeh, Hanieh-Sadat Ejtahed, Farshad Sharifi

**Affiliations:** ^1^ Research Center for Prevention of Cardiovascular Diseases, Endocrinology & Metabolism, Institute of Endocrinology Metabolism, Iran University of Medical Sciences, Tehran, Iran, iums.ac.ir; ^2^ Endocrinology and Metabolism Research Center, Endocrinology and Metabolism Clinical Sciences Institute, Tehran University of Medical Sciences, Tehran, Iran, tums.ac.ir; ^3^ Metabolomics and Genomics Research Center, Endocrinology and Metabolism Molecular-Cellular Sciences Institute, Tehran University of Medical Sciences, Tehran, Iran, tums.ac.ir; ^4^ Elderly Health Research Center, Endocrinology and Metabolism Population Sciences Institute, Tehran University of Medical Sciences, Tehran, Iran, tums.ac.ir; ^5^ Geriatric Health Research Center, Birjand University of Medical Sciences, Birjand, Iran, bums.ac.ir; ^6^ School of Health, Birjand University of Medical Sciences, Birjand, Iran, bums.ac.ir; ^7^ Obesity and Eating Habits Research Center, Endocrinology and Metabolism Clinical Sciences Institute, Tehran University of Medical Sciences, Tehran, Iran, tums.ac.ir; ^8^ Department of Gerontology, School of Rehabilitation, Tehran University of Medical Sciences, Tehran, Iran, tums.ac.ir

**Keywords:** Birjand longitudinal aging study, muscle mass, pain, sarcopenia

## Abstract

**Background:**

Sarcopenia, characterized by the loss of muscle mass and strength, is a prevalent geriatric condition associated with increased morbidity and healthcare burden. Understanding its multifactorial etiology is essential for effective prevention and management.

**Objectives:**

This study aimed to investigate the association between chronic pain and sarcopenia and to identify demographic, nutritional, and functional factors associated with sarcopenia in a cohort of older Iranian adults.

**Methods:**

A cross‐sectional analysis was conducted using baseline data from 1344 participants aged ≥ 60 years from the Birjand Longitudinal Aging Study (BLAS). Participants were classified into four groups based on the European Working Group on Sarcopenia in Older People‐version 2 (EWGSOP2) criteria: robust, probable sarcopenia, sarcopenia, and severe sarcopenia. Pain was assessed using the brief pain inventory (BPI), with pain severity score and pain interference score (BPI9) as primary variables. Multivariable and multinomial logistic regression models adjusted for socioeconomic, nutritional, and functional covariates were used to assess associations.

**Results:**

In logistic regression analyses, pain interference score was significantly associated with higher odds of sarcopenia across all models, including the fully adjusted model (OR = 1.24, 95% CI: 1.11–1.39, *p* < 0.001), whereas pain severity score was not associated with higher odds of sarcopenia in binary models (*p* > 0.05). In multinomial logistic regression, pain interference score demonstrated a graded association with sarcopenia severity, with the strongest effect observed for severe sarcopenia (final model RRR = 1.32, 95% CI: 1.15–1.52, *p* < 0.001) and a moderate effect for probable sarcopenia (RRR = 1.13, 95% CI: 1.05–1.22, *p* = 0.003). The association between pain severity and sarcopenia severity was not significant in final adjusted model. Sensitivity analyses using an alternative sarcopenia definition confirmed these findings.

**Conclusion:**

Pain interference, rather than pain severity, is significantly associated with sarcopenia. These findings highlight the importance of addressing pain‐related functional limitations in efforts to prevent and manage sarcopenia, particularly in aging populations within low‐ and middle‐income countries.

## 1. Introduction

Sarcopenia is characterized by the gradual loss of muscle mass and strength [1]. By the time individuals reach their 50s, muscle mass and strength decline annually at rates of 1%–2% and 1.5%–5%, respectively [2]. According to a recent analysis conducted on 58,404 individuals, the prevalence of sarcopenia was 10% globally [3]. Sarcopenia increases the risk of falls, disability, postsurgical complications, and mortality [4]. Annually $257.1 million budget is allocated to sarcopenic patients in Iran [5]. Since sarcopenia can be reversed with proper interventions, it is crucial to identify the risk factors associated with this condition [6]. There is some evidence that pain is one of them. There are some underlying mechanisms in the relationship between pain and sarcopenia, for instance, systemic inflammation and the release of cytokines, especially IL‐6 and TNF‐α, can accelerate sarcopenia [7, 8]. According to the fear‐avoidance model, the experience of musculoskeletal pain generates a fear of further pain, leading to avoidance behaviors. This avoidance can result in immobilization and disuse of affected areas, which are known as risk factors for sarcopenia [9, 10]. Malnourishment and genetic factors are among other common risk factors for both pain and sarcopenia [11–13].

The relationship between pain and sarcopenia remains controversial. A 10‐year longitudinal study of 4102 participants found that severe pain is significantly associated with sarcopenia, regardless of the pain site [14]. Similarly, a survey of 14,585 elderly individuals in low‐ and middle‐income countries (LMICs) showed a positive correlation between pain levels and sarcopenia [10]. Another study observed that individuals with persistent chronic or intrusive pain over 5 years had a higher incidence of sarcopenia [15]. On the other hand, a cross‐sectional study found no association between low back pain and sarcopenia [16].

Given the significant burden that sarcopenia places on healthcare systems, along with the high prevalence of pain among older adults and the increasing life expectancy [17, 18], it is essential to take proactive measures to enhance the quality of life for the elderly. Therefore, for the first time, we aimed to investigate the association between pain and sarcopenia in a community‐based study of older adults in Iran.

## 2. Methods

### 2.1. Ethical Considerations

This study was conducted as part of the Birjand Longitudinal Aging Study (BLAS) in Birjand, Iran. Ethical approval of BLAS was obtained from the Ethics Committees of Birjand University of Medical Sciences (Ethical Code: IR.BUMS.Rec.1397.282) and Endocrinology and Metabolism Research Institute, Tehran University of Medical Sciences (Ethical Code: IR.TUMS.EMRI.REC.1396.00158). The current study was also approved by the Ethics Committee of Endocrinology and Metabolism Research Institute, Tehran University of Medical Sciences (Ethical Code: IR.TUMS.EMRI.REC.1403.123). All procedures adhered to the Declaration of Helsinki’s guidelines. Informed consent was obtained from all participants; for those with cognitive impairments (Abbreviated Mental Test Score < 7), consent was provided by a close relative or legal guardian. For illiterate participants, the consent form was read aloud by a trusted individual, and consent was indicated via fingerprint.

### 2.2. Study Design and Population

This cross‐sectional analysis utilized baseline data from BLAS, a cohort study initiated in 2018 focusing on community‐dwelling older adults aged 60 years and above in Birjand, Iran [19]. The primary objective of BLAS is to investigate the prevalence and risk factors associated with geriatric syndromes. Participants were selected through multistage cluster random sampling based on postal codes, encompassing both urban and rural areas. Inclusion criteria were: age ≥ 60 years and ability to participate in the study. Exclusion criteria included being bedridden, severe cognitive impairment (scores of less than three measured with abbreviated mental test score), unable to communicate, or a life expectancy of less than 6 months. Data collection was performed through standardized face‐to‐face interviews conducted by trained researchers, ensuring methodological consistency. Data were recorded using the “Digit” online software platform, which incorporated automated validation to ensure data integrity.

### 2.3. Variables and Measurements

#### 2.3.1. Pain Assessment

Pain was evaluated using the culturally adapted and validated Iranian version of the brief pain inventory (BPI). The BPI assesses pain in terms of location, intensity, relief measures, and interference with daily activities. Pain severity score was derived from the mean of items 3–6 (worst, least, average, and current pain in the past week), each scored on a 0–10 numeric rating scale. Pain interference was assessed with the BPI‐9 score, which includes seven items evaluating how much pain disrupts general activity, walking ability, work, mood, enjoyment of life, social relationships, and sleep. Each item is rated on a 10‐point Likert scale from 0 (“does not interfere”) to 10 (“completely interferes”), and the overall pain interference score is calculated as the mean of these seven items [20].

#### 2.3.2. Sarcopenia Assessment

Sarcopenia was diagnosed based on the criteria established by the European Working Group on Sarcopenia in Older People, version 2 (EWGSOP2) [1]. The assessment included the following:

• **Muscle Mass**: Measured using bioelectrical impedance analysis (BIA), with low muscle mass defined as an appendicular skeletal muscle mass index (ASM/height^2^) below 7.0 kg/m^2^ for men and 5.5 kg/m^2^ for women. All BIA assessments were conducted under standardized conditions. Participants were measured in a fasted state and instructed to avoid strenuous physical activity for at least 12 h prior to assessment, and the same device model was used consistently throughout the study. Additionally, the interpretation of BIA‐derived indices was performed with reference to the device’s established population‐specific norms to improve clarity, transparency, and reproducibility.
• **Muscle Strength**: Assessed via handgrip strength using a calibrated dynamometer; low muscle strength was defined as < 27 kg for men and < 16 kg for women.
• **Physical Performance**: Evaluated using the gait speed (cutoff ≤ 0.8 m/s), the short physical performance battery (SPPB) (cutoff ≤ 8 point score), and the timed up and go (TUG) test, with a cutoff time of > 20 s indicating poor performance.

Participants meeting the criteria for low muscle strength were classified as probable sarcopenia. Participants with low muscle strength and low muscle mass were classified as confirmed sarcopenia, and participants with low muscle strength, low muscle mass, and low physical performance were classified as severe sarcopenia.

#### 2.3.3. Covariates

• **Demographic variables**: Data on sex, age, and education level (years of formal education) were collected through structured interviews.
• **Anthropometric measurements**: Height and weight were measured to calculate body mass index (BMI), categorized according to WHO standards: underweight (< 18.5 kg/m^2^), normal weight (18.5–24.99 kg/m^2^), overweight (25–29.99 kg/m^2^), and obese (≥ 30 kg/m^2^).
• **Smoking status**: Classified as current smoker, former smoker, or never smoked, based on self‐report.
• **Physical activity**: Evaluated using the Longitudinal Aging Study Amsterdam Physical Activity Questionnaire (LAPAQ) [21]. The questionnaire assesses physical activity at work and during leisure time. Participants activities’ frequency, duration, and intensity in the past 2 weeks were recorded. Participants with no physical activity were considered inactive, while others with at least once per 2 weeks were considered physically active.
• **Wealth quintile**: Determined based on self‐reported assets and income, categorizing participants into five socioeconomic groups. The assets included refrigerator, vacuum cleaner, microwave oven, washing machine, dish machine, water purifier, LED television, smartphone, automobile, and house. Principal component analysis (PCA) was conducted to measure wealth index.
• **Depressed mood:** Assessed using the Patient Health Questionnaire‐9 (PHQ‐9) [22]. Scores of 10 or higher were considered as depressed mood.
• **Sleep quality:** Assessed using selected questions of the Pittsburg Sleep Quality Index (PSQI).
• **Polypharmacy:** Defined as taking five or more medications daily.

### 2.4. Statistical Analysis

Data analysis was performed using Stata version 17 (StataCorp, College Station, TX, USA). Descriptive statistics were calculated for all variables. Continuous variables were presented as means with standard deviations, and categorical variables as frequencies with percentages. Comparisons between groups were conducted using one‐way analysis of variance (ANOVA) for continuous variables and chi‐square tests for categorical variables.

In our primary logistic regression analysis, we categorized participants into two groups: those with sarcopenia (including individuals diagnosed with sarcopenia and severe sarcopenia) and those without sarcopenia (comprising robust individuals and those with probable sarcopenia). This primary analysis utilized a more conservative case definition to reduce potential misclassification. This binary classification was utilized to assess the association between pain, as measured by BPI, and the presence of sarcopenia.

To evaluate the robustness of our findings, we conducted a sensitivity analysis with an alternative classification scheme. In this analysis, adopting a broader definition aligned with the EWGSOP2 conceptual framework, the sarcopenia group encompassed participants with probable sarcopenia, sarcopenia, and severe sarcopenia, while the nonsarcopenia group included only robust individuals. This approach allowed us to examine whether the observed associations persisted under different diagnostic thresholds.

**FIGURE 1:**
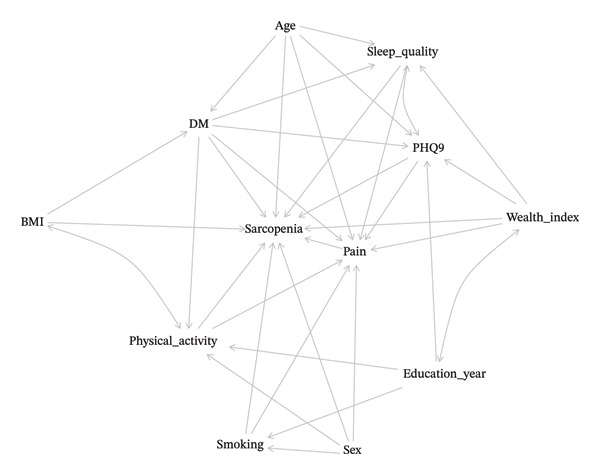
Directed acyclic graph (DAG) illustrating the assumed causal relationships among pain (exposure), sarcopenia (outcome), and candidate covariates.

To examine the association between pain (BPI scores) and sarcopenia, multivariate and multinominal logistic regression models were employed. Sarcopenia’s status served as the dependent variable, and BPI scores were the primary independent variables. Covariate selection for the multivariable models was informed by a directed acyclic graph (DAG) developed using DAGitty (Figure 1) [23]. The final model included 9 covariates with 136 outcome events, yielding an events‐per‐variable (EPV) ratio of approximately 15, above the commonly recommended minimum of 10 [24]. Multicollinearity among covariates in the final model was assessed using variance inflation factors (VIF). All VIF values were below 1.3 (mean VIF = 1.13), indicating no concerning multicollinearity.

## 3. Results

A total of 1344 participants were classified into four groups: Severe Sarcopenia (*n* = 72), Sarcopenia (*n* = 64), Probable Sarcopenia (*n* = 417), and Robust (*n* = 791). Most participants in the Robust group were aged 60–69 years (519 individuals, 65.61%), while the Severe Sarcopenia group had a higher proportion (36 individuals) of individuals aged 80 years and older (*p*‐value< 0.001). There was a roughly equal gender distribution across the groups, although females made up the majority in the Severe Sarcopenia (54 females, 75%) and Sarcopenia (32 females, 50%) groups (*p*‐value = 0.001). The education level varied considerably across groups, with a high percentage of individuals in the Severe Sarcopenia group being illiterate (60 individuals, 83.33%), compared to only 35.15% in the Robust group. The Robust group had a notably higher proportion of individuals with higher education (82 individuals, 10.37%), while those with Severe Sarcopenia had very few with a high school diploma or higher (*p*‐value< 0.001). BMI classification revealed that 62.50% of participants with Severe Sarcopenia had an ideal body weight (45 individuals), while a notable portion (16.67%) were in the low body weight category. The Robust group had a higher prevalence of overweight individuals (327 participants, 41.34%, *p*‐value < 0.001). Additional baseline characteristics are summarized in Table 1.

**TABLE 1:** Baseline characteristics of the participants.

Variables	Severe sarcopenia	Sarcopenia	Probable sarcopenia	Robust	*p*‐value
Total population	72 (5.36%)	64 (4.76%)	417 (31.03%)	791 (58.85%)	
Age^∗^	78.9 ± 7.6	72.6 ± 7.6	71.3 ± 8.2	67.8 ± 6.1	< 0.001
60–69 years	7 (9.72%)	27 (42.19%)	203 (48.68%)	519 (65.61%)	
70–79 years	29 (40.28%)	25 (39.06%)	130 (31.18%)	236 (29.84%)	
80+ years	36 (50%)	12 (18.75%)	84 (20.14%)	36 (4.55%)	
Gender					0.001
Male	18 (25%)	32 (50%)	201 (48.2%)	396 (50.06%)	
Female	54 (75%)	32 (50%)	216 (51.8%)	395 (49.94%)	
BMI	22.84 ± 5.06	21.1 ± 4.01	27.30 ± 5.21	26.70 ± 5.13	< 0.001
Ideal body weight	45 (62.5%)	35 (54.7%)	142 (34.1%)	263 (33.2%)	
Low body weight	12 (16.7%)	20 (31.3%)	6 (1.4%)	25 (3.2%)	
Overweight	9 (12.5%)	8 (12.5%)	167 (40.0%)	327 (41.3%)	
Obese	6 (8.3%)	1 (1.6%)	102 (24.5%)	176 (22.3%)	
Education year^∗^	0.9 ± 2.3	2.1 ± 3.8	3.3 ± 4.5	5.4 ± 5.3	< 0.001
Illiterate	60 (83.33%)	45 (70.31%)	223 (53.48%)	278 (35.15%)	
Primary school	10 (13.89%)	13 (20.31%)	136 (32.61%)	263 (33.25%)	
High school	1 (1.39%)	2 (3.13%)	13 (3.12%)	59 (7.46%)	
Diploma	1 (1.39%)	1 (1.56%)	22 (5.28%)	109 (13.78%)	
Academic	0 (0%)	3 (4.69%)	23 (5.52%)	82 (10.37%)	
Wealth					< 0.001
First quintile	37 (51.39)	23 (35.94)	93 (22.30)	114 (14.41)	
Second quintile	15 (20.83)	17 (26.56)	98 (23.50)	136 (17.19)	
Third quintile	10 (13.89)	13 (20.31)	95 (22.78)	151 (19.09)	
Fourth quintile	4 (5.56)	6 (9.38)	58 (13.91)	201 (25.41)	
Fifth quintile					

Abbreviations: BMI, body mass index; MNA, mini nutritional assessment.

^∗^Continuous data presented in mean (SD).

Regarding physical activity, 54.17% of participants with Severe Sarcopenia (39 individuals) were inactive, while 55.44% of the Robust group (438 individuals) were active (*p*‐value = 0.002). Smoking status did not show significant differences across groups, with most participants in all groups being nonsmokers (90.28% in Severe Sarcopenia, 91.15% in Robust, *p*‐value = 0.255). Sleep quality was generally good across groups, with 76.39% of Severe Sarcopenia participants (55 individuals) reporting good sleep quality, like 76.11% of the Robust group (602 individuals, *p*‐value = 0.629). The prevalence of depressive symptoms, assessed using the PHQ‐9, was significantly higher in the Severe Sarcopenia group, with 34.72% of participants (25 individuals) reporting depressive symptoms, including 4.17% (3 individuals) with severe depressive symptoms. In the Robust group, 83.12% (655 individuals) had no depressive symptoms, and only 1.40% (11 individuals) had severe depressive symptoms (*p*‐value < 0.001). Finally, the prevalence of diabetes was higher in the Probable Sarcopenia group (125 individuals, 29.98%) compared to the other groups. In the Severe Sarcopenia group, 15.28% (11 individuals) were diagnosed with diabetes, while 27.81% of Robust participants (220 individuals) had diabetes, with the majority (72.19%) being diabetes‐free (*p*‐value = 0.003). More information is presented in Table 2.

**TABLE 2:** Comorbidities and lifestyle of participants.

Variables	Severe sarcopenia	Sarcopenia	Probable sarcopenia	Robust	*p*‐value
Physical activity					0.002
Inactive	39 (54.17%)	34 (53.13%)	232 (55.64%)	352 (44.56%)	
Active	33 (45.83%)	30 (46.88%)	185 (44.36%)	438 (55.44%)	
Current smoking					0.255
Yes	7 (9.72%)	9 (14.06%)	29 (6.95%)	70 (8.85%)	
No	65 (90.28%)	55 (85.94%)	388 (93.05%)	721 (91.15%)	
Sleep quality^∗^	1.2 ± 0.7	1.1 ± 0.5	1.2 ± 0.6	1.2 ± 0.6	0.629
Good	55 (76.39%)	53 (82.81%)	314 (75.30%)	602 (76.11%)	
Bad	17 (23.61%)	11 (17.19%)	103 (24.70%)	189 (23.89%)	
PHQ9^∗^	7.3 ± 5.7	5.4 ± 4.7	6.1 ± 5.2	4.9 ± 5.0	< 0.001
Not depressed mood	47 (65.28%)	54 (84.38%)	317 (76.20%)	655 (83.12%)	
Depressed mood	25 (34.72%)	10 (15.63%)	99 (23.80%)	133 (16.88%)	
Pain severity score^∗^	4.34 ± 2.15	3.64 ± 2.53	4.13 ± 2.32	3.60 ± 2.32	< 0.001
Pain interference score (BPI‐9)^∗^	4.41 ± 2.52	2.68 ± 2.05	3.05 ± 2.19	2.26 ± 1.71	< 0.001
Diabetes					0.003
Yes	11 (15.28%)	8 (12.50%)	125 (29.98%)	220 (27.81%)	
No	61 (84.72%)	56 (87.50%)	292 (70.02%)	571 (72.19%)	

*Note:* PHQ9, Patient Health Questionnaire.

^∗^Continuous data presented in mean (SD).

### 3.1. Logistic Regression Models

**FIGURE 2:**
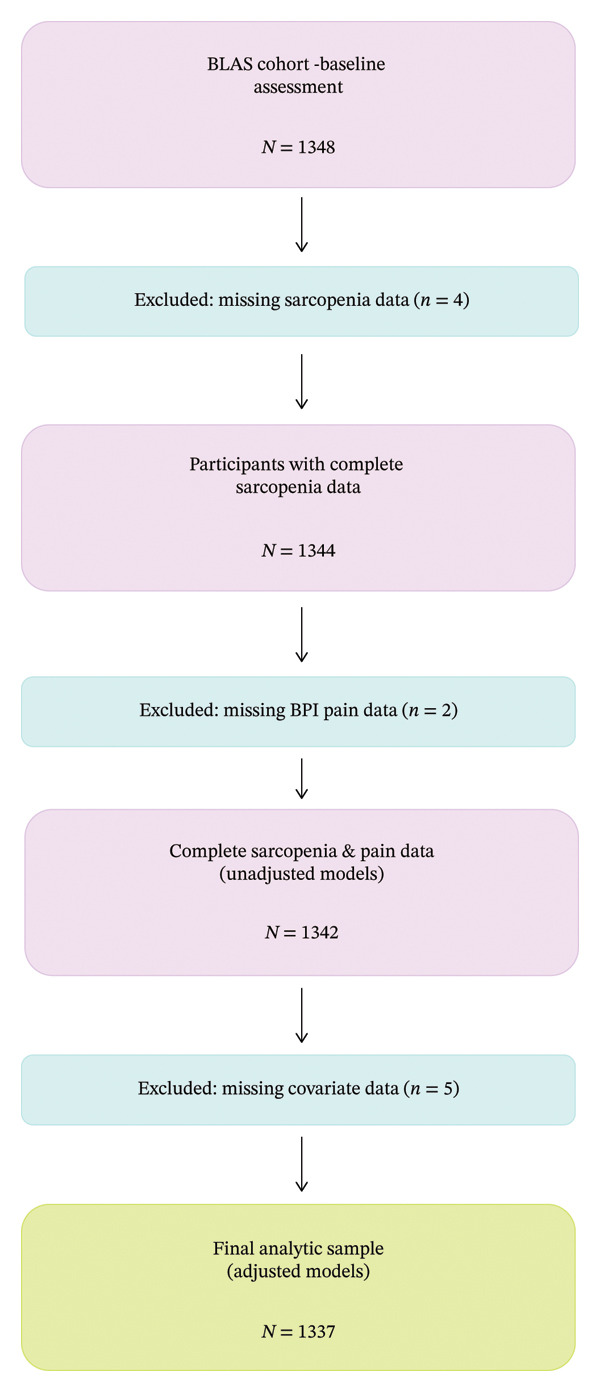
Participant flow diagram. From 1348 participants in the BLAS cohort baseline assessment, 4 persons were excluded due to missing sarcopenia data, 2 due to missing BPI pain assessment, and 5 due to missing covariate data, yielding a final analytic sample of 1337 participants for the adjusted models.

The final analytic sample for adjusted models comprised 1337 participants with complete data on sarcopenia, pain measures, and all covariates (Figure 2). Pain severity score was not associated with sarcopenia in the unadjusted model (OR = 1.10, 95% CI: 1.05–1.15, *p* < 0.001), and was still nonsignificant after further adjustment for socioeconomic status, sleep quality, depression, diabetes, smoking, and physical activity (final model) (OR = 0.94, 95% CI: 0.86–1.04, *p* = 0.227). Similarly, in the fully adjusted model (Model 4), pain severity score showed no significant association with sarcopenia (OR = 1.01, 95% CI: 0.91–1.11, *p* = 0.890).

In contrast, pain interference score was significantly associated with sarcopenia across all four models. In the unadjusted model, each unit increase in BPI‐9 was associated with a 24% increase in the odds of sarcopenia (OR = 1.24, 95% CI: 1.15–1.33, *p* < 0.001). This association remained robust and statistically significant after sequential adjustment for age and sex (Model 2: OR = 1.10, 95% CI: 1.0.1–1.19, *p* = 0.033), additional lifestyle and health covariates (final model: OR = 1.12, 95% CI: 1.01–1.24, *p* = 0.033), and in the fully adjusted model (Model 4: OR = 1.24, 95% CI: 1.11–1.39, *p* < 0.001). Detailed results are presented in Table 3.

**TABLE 3:** Association between pain severity score (BPIS) and pain interference score (BPI‐9) and sarcopenia risk: Binary logistic regression models.

Model	Pain severity score (BPIS)		Pain interference score (BPI‐9)	
	Or (95% CI)	*p*‐value	Or (95% CI)	*p*‐value
Model 1	1.04 (0.97–1.13)	0.278	1.24 (1.15–1.33)	**< 0.001**
Model 2	0.97 (0.89–1.05)	0.449	1.10 (1.01–1.19)	**0.033**
Model 3 (final model)	0.94 (0.86–1.04)	0.227	1.12 (1.01–1.24)	**0.033**
Model 4 (fully adjusted model)	1.01 (0.91–1.11)	0.890	1.24 (1.11–1.39)	**< 0.001**

*Note:* Model 1: Crude (unadjusted); parameters (df): 1; EPV: 136. Model 2: Adjusted for sex and age; parameters (df): 3; EPV: 45.3. Model 3: Adjusted for sex, age, wealth index, sleep quality, PHQ9, DM, smoking, and physical activity; parameters (df): 9; EPV: 15.1; all covariates in Model 3 had VIF values < 1.3 (mean VIF = 1.13), indicating no concerning collinearity. Model 4: Adjusted for sex, age, BMI, education, wealth index, sleep quality, PHQ9, DM, smoking, and physical activity; parameters (df): 11; EPV: 12.4. PHQ = Patient Health Questionnaire (depression score). Bold values indicate statistical significance (*p* < 0.05).

Abbreviations: CI = confidence interval, DM = diabetes mellitus, OR = odds ratio, PA = physical activity.

### 3.2. Multinominal Logistic Regression Analysis

Pain severity score demonstrated significant associations with probable sarcopenia only in the minimally adjusted models. In the unadjusted model, each unit increase in BPIS was associated with an 11% increase in the relative risk of probable sarcopenia (Model 1: RRR = 1.11, 95% CI: 1.05–1.17, *p* < 0.001), which attenuated slightly but remained significant after adjustment for age and sex (Model 2: RRR = 1.09, 95% CI: 1.03–1.15, *p* = 0.003). However, this association was no longer significant after further adjustment for socioeconomic, lifestyle, and clinical covariates (final model: RRR = 1.04, 95% CI: 0.97–1.10, *p* = 0.262; Model 4: RRR = 1.02, 95% CI: 0.96–1.09, *p* = 0.468). For severe sarcopenia, pain severity score was significantly associated only in the unadjusted model (Model 1: RRR = 1.15, 95% CI: 1.04–1.28, *p* = 0.009), with the association attenuating to the null after age and sex adjustment (Model 2: RRR = 1.03, 95% CI: 0.91–1.16, *p* = 0.641) and remaining nonsignificant in all subsequent models. Pain severity score was not significantly associated with confirmed sarcopenia in any model (Table 4).

**TABLE 4:** Association between pain severity score and pain interference score with sarcopenia severity: Multinomial logistic regression models.

Model	Outcome	Pain severity score		Pain interference score (BPI‐9)	
		RRR (95% CI)	*p*‐value	RRR (95% CI)	*p*‐value
Model 1	Probable sarcopenia	**1.11 (1.05–1.17)**	**< 0.001**	**1.23 (1.16–1.31)**	**< 0.001**
	Sarcopenia	1.01 (0.90–1.13)	0.896	1.13 (1.00–1.29)	0.059
	Severe sarcopenia	**1.15 (1.04–1.28)**	**0.009**	**1.53 (1.39–1.70)**	**< 0.001**

Model 2	Probable sarcopenia	**1.09 (1.03–1.15)**	**0.003**	**1.18 (1.11–1.26)**	**< 0.001**
	Sarcopenia	0.98 (0.87–1.11)	0.768	1.06 (0.93–1.22)	0.380
	Severe sarcopenia	1.03 (0.91–1.16)	0.641	**1.31 (1.16–1.47)**	**< 0.001**

Model 3 (final model)	Probable sarcopenia	1.04 (0.97–1.10)	0.262	**1.13 (1.05–1.22)**	**0.002**
	Sarcopenia	0.94 (0.83–1.07)	0.386	1.05 (0.89–1.23)	0.567
	Severe sarcopenia	0.98 (0.85–1.12)	0.748	**1.32 (1.15–1.52)**	**< 0.001**

Model 4 (fully adjusted model)	Probable sarcopenia	1.02 (0.96–1.09)	0.468	**1.13 (1.04–1.22)**	**0.003**
	Sarcopenia	1.01 (0.89–1.16)	0.832	1.15 (0.98–1.36)	0.091
	Severe sarcopenia	1.02 (0.89–1.18)	0.732	**1.46 (1.25–1.70)**	**< 0.001**

*Note:* Reference outcome category: Robust. Model 1: Crude (unadjusted). Model 2: Adjusted for sex and age. Model 3: Adjusted for sex, age, wealth index, and sleep quality, PHQ9, DM, smoking, and physical activity. Model 4: Adjusted for sex, age, BMI, education, wealth index, sleep quality, PHQ9, DM, smoking, and physical activity. PHQ = Patient Health Questionnaire (depression score). Bold values indicate statistical significance (*p* < 0.05).

Abbreviations: CI = confidence interval, DM = diabetes mellitus, PA = physical activity, RRR = relative risk ratio.

Pain interference score (BPI‐9) demonstrated a consistent, robust, and statistically significant association with both probable and severe sarcopenia across all four models. For probable sarcopenia, BPI‐9 was associated with a 23% increase in relative risk in the unadjusted model (Model 1: RRR = 1.23, 95% CI: 1.16–1.31, *p* < 0.001), and this association persisted with little attenuation across all levels of adjustment, including the fully adjusted model (Model 4: RRR = 1.13, 95% CI: 1.04–1.22, *p* = 0.003). For severe sarcopenia, the association was notably stronger in magnitude: BPI‐9 was associated with a 53% increase in relative risk in the unadjusted model (Model 1: RRR = 1.53, 95% CI: 1.39–1.70, *p* < 0.001) and a 32% increase in the final model (Model 3: RRR = 1.32, 95% CI: 1.15–1.52, *p* < 0.001). For confirmed sarcopenia, BPI‐9 showed a borderline significant association in the unadjusted model (Model 1: RRR = 1.13, 95% CI: 1.00–1.29, *p* = 0.059), which did not reach statistical significance after covariate adjustment (Model 2: RRR = 1.06, 95% CI: 0.93–1.22, *p* = 0.380; Model 3: RRR = 1.05, 95% CI: 0.89–1.23, *p* = 0.567; Model 4: RRR = 1.15, 95% CI: 0.98–1.36, *p* = 0.091).

### 3.3. Sensitivity Analysis

In our logistic models, we initially grouped robust and probable sarcopenic patients as nonsarcopenic, and sarcopenic and severely sarcopenic patients as sarcopenic. However, for the sensitivity analysis, we redefined sarcopenia to encompass probable sarcopenia, sarcopenia, and severe sarcopenia to test the robustness of our findings across different definitional criteria. Table 5 represents the results of a sensitivity analysis examining the factors associated with sarcopenia across different models. Following the recategorization of probable sarcopenia as sarcopenia, the final logistic regression model indicated different relationships for pain severity score and BPI‐9 with sarcopenia. In the final model, pain severity score showed no significant association (OR = 1.02, 95% CI: 0.96–1.08, *p* = 0.546), while BPI‐9 was significantly and positively associated with sarcopenia (OR = 1.15, 95% CI: 1.06–1.23, *p* < 0.001).

**TABLE 5:** Sensitivity analysis: Association between pain severity score (BPIS) and pain interference score (BPII) with sarcopenia; odds ratios from binary logistic regression models.

Model	Pain severity (BPIS)		Pain interference (BPI‐9)	
	OR (95% CI)	*p*‐value	OR (95% CI)	*p*‐value
Model 1	**1.10 (1.05–1.15)**	**0.000**	**1.26 (1.19–1.33)**	**< 0.001**
Model 2	**1.07 (1.02–1.13)**	**0.011**	**1.18 (1.11–1.26)**	**< 0.001**
Model 3 (final model)	1.02 (0.96–1.08)	0.546	**1.15 (1.06–1.23)**	**< 0.001**
Model 4	1.01 (0.96–1.08)	0.639	**1.16 (1.07–1.24)**	**< 0.001**

*Note:* Model 1: Crude (unadjusted). Model 2: Adjusted for sex and age. Model 3: Adjusted for sex, age, wealth index, sleep quality, PHQ9, DM, smoking, and physical activity. Model 4: Adjusted for sex, age, BMI, education, wealth index, sleep quality, PHQ9, DM, smoking, and physical activity. BPIS = brief pain inventory – pain severity; BPII = brief pain inventory – pain interference; PHQ = Patient Health Questionnaire (depression score). Bold values indicate statistical significance (*p* < 0.05).

Abbreviations: CI = confidence interval, DM = diabetes mellitus, OR = odds ratio, PA = physical activity.

## 4. Discussion

This cross‐sectional analysis of older Iranian adults from the BLAS cohort revealed that chronic pain interference, measured by the BPI‐9, was associated with higher odds of sarcopenia in the crude binary analysis. A one‐point increase in the pain interference score in the final model corresponded to a 12% increase in the odds of sarcopenia, whereas pain severity was not associated with sarcopenia in the final model. Pain interference score, but not pain severity score, was progressively more strongly associated with more advanced stages of the condition, with the greatest risk observed for severe sarcopenia. Interestingly, although pain severity was not significantly associated with sarcopenia after full adjustment, the partially adjusted models demonstrated a modest inverse trend, suggesting that pain intensity alone may not reflect the functional and behavioral consequences of chronic pain that are more directly linked to muscle decline. This observation further supports the concept that pain‐related disability and activity limitation, rather than pain intensity itself, may be the more clinically relevant determinants of sarcopenia risk [25, 26]. To our knowledge, this is one of the first studies to distinguish the functional impact of pain from pain severity in relation to sarcopenia risk.

Our results are consistent with a growing body of international evidence. A recent meta‐analysis found that chronic pain increases sarcopenia risk by 52%, with stronger associations observed in low‐income countries where access to pain management and rehabilitation is often limited [27]. Another large study conducted in LMICs identified mobility impairment and sedentary behavior as key mediators in the relationship between chronic pain and sarcopenia [10]. Similarly, in a Greek cohort, 44% of sarcopenic older adults reported chronic pain, and the presence of chronic pain was significantly associated with sarcopenia, alongside polypharmacy and depressive symptoms [28]. Chronic pain can initiate a cycle of reduced physical activity, muscle disuse, and functional decline, which over time accelerates sarcopenia [25]. Within the CHARLS longitudinal cohort, which encompassed 12,788 Chinese participants aged 45 or more, persistent pain was significantly associated with future declines in grip strength and physical performance, though not with muscle mass alone [26]. This supports the recent EWGSOP2 emphasis on functional criteria rather than purely anatomical ones and validates our result that pain interference, rather than intensity, is the more clinically salient predictor [1]. Clinically, this distinction carries important implications. While pain severity is often the focus of treatment, interference, such as difficulty walking, self‐caring, or engaging in activities, may be a more relevant therapeutic target in sarcopenia prevention. Strategies focusing on preserving mobility despite chronic pain, including timely analgesia, physical therapy, and the use of assistive devices, may help mitigate muscle loss and prevent functional decline [25].

Depression also showed a notable pattern in our study. It was more prevalent among participants with severe sarcopenia. This reflects a complex relationship where depression, chronic pain, and physical inactivity intersect. A meta‐analysis reported that the prevalence of depression in sarcopenic individuals is approximately 28%, with an adjusted odds ratio of 1.57 between sarcopenia and depression [29]. Similarly, Gao et al. identified late‐life depression as an independent factor associated with sarcopenia [30]. One explanation is that chronic pain may partially mediate the relationship between sarcopenia and depression. Zhou et al. demonstrated that depressive symptoms explained part of the effect of persistent musculoskeletal pain on incident sarcopenia among older Chinese adults [26]. In other words, chronic pain can lead to both depressed mood and muscle decline, making depression a downstream factor in some cases. Conversely, depression may also contribute to sarcopenia by reducing appetite, impairing nutrition, and lowering motivation for physical activity [25]. However, unmeasured factors like social isolation or pain medication use might have confounded this relationship.

A recent Thai meta‐analysis reported that low BMI was the strongest predictor of sarcopenia, increasing the odds nearly 9‐fold [31]. While some research suggests that obesity may offer protective effects on grip strength, particularly in men, the evidence is inconsistent [32]. In our study, underweight status was clearly detrimental, whereas obesity conferred no apparent protective effect. This aligns with studies showing that BMI alone may be an inadequate surrogate for sarcopenia, and that sarcopenic obesity (coexisting low muscle mass with excess fat) poses unique risks [33].

Older adults with lower levels of physical activity had significantly increased odds of sarcopenia in our study. Hämäläinen et al. similarly found that insufficient physical activity, measured both via self‐report and accelerometry, was associated with probable sarcopenia in a European cohort [34]. A meta‐analysis of risk factors in community‐dwelling elders confirmed that physical inactivity is consistently associated with increased sarcopenia risk [30]. These findings underscore the importance of promoting regular, safe movement in older adults as a preventive strategy, even in the presence of chronic pain.

Several sociodemographic variables also contributed to sarcopenia risk in our sample, highlighting broader determinants of health in later life. Unsurprisingly, advanced age was strongly associated with sarcopenia, a near‐universal finding given its degenerative nature. Interestingly, we observed higher sarcopenia prevalence among women, a result consistent with some LMIC studies but contrary to those from higher‐income settings, where men often show greater prevalence due to greater absolute muscle loss [31]. A previous meta‐analysis found no definitive sex difference in sarcopenia risk, likely due to heterogeneity across studies [30].

From a public health standpoint, our findings have clear implications. LMICs such as Iran are experiencing rapid population aging without corresponding expansion in geriatric care infrastructure. Sarcopenia remains underdiagnosed and is not widely prioritized in national health policies. Our findings suggest that simple, low‐cost tools—such as grip strength assessments and the BPI‐9 questionnaire—can help identify at‐risk individuals in primary care and community settings. Integrating sarcopenia screening into routine health services could enhance early detection and facilitate timely intervention.

Multidisciplinary, integrated approaches are increasingly advocated. Grosman and Kalichman have proposed comprehensive frameworks that integrate resistance training, nutritional counseling, and pain management to address the interconnected burdens of sarcopenia and chronic musculoskeletal pain [25].

## 5. Limitations

First of all, the study’s cross‐sectional nature precludes causal inference and probability of reverse causation cannot be excluded. Second, data were drawn exclusively from the BLAS cohort, which may limit generalizability to other Iranian regions or LMIC settings with different sociocultural or healthcare infrastructures. Third, pain interference, physical activity, and wealth quintile rely on self‐report, introducing recall and social desirability biases. Fourth, unmeasured factors—such as inflammatory biomarkers, medication adherence, and psychosocial stress—may confound the observed associations. Fifth, exclusion of bedridden or severely cognitively impaired individuals may underestimate the true burden of sarcopenia and high‐impact pain in the most vulnerable subgroups. Lastly, our study did not assess the impact of pain management strategies (e.g., analgesics, physiotherapy) on sarcopenia risk. Exploring whether effective pain control mitigates sarcopenia could guide clinical interventions.

## 6. Conclusion

In conclusion, this study provides a detailed examination of factors associated with sarcopenia in older adults and highlights the critical distinction between pain severity and pain interference scores. We found that chronic pain that disrupts daily functioning is significantly associated with higher odds of sarcopenia, whereas pain severity alone, in the absence of functional limitations, is not. This insight underscores the importance of targeting pain‐related disability in efforts to preserve muscle strength and function in aging populations. In rapidly aging LMICs such as Iran, early detection and integrated, cost‐effective prevention strategies are essential to supporting independence and well‐being in later life. Future studies should incorporate serial assessments of both pain interference and muscle health to clarify temporal and causal relationships; evaluate the effects of targeted pain management interventions on muscle mass and function; and explore underlying neuro‐immune mechanisms by measuring inflammatory biomarkers alongside patient‐reported outcomes.

## Funding

No funding was received for this manuscript.

## Conflicts of Interest

The authors declare no conflicts of interest.

## Data Availability

The data that support the findings of this study are available upon request from the corresponding author. The data are not publicly available due to privacy or ethical restrictions.
